# Effect of two whitening agents on the color of composite dental restorations

**DOI:** 10.4317/jced.55450

**Published:** 2019-01-01

**Authors:** José Amengual-Lorenzo, José-María Montiel-Company, Carlos Bellot-Arcís, Carlos Labaig-Rueda, María-Fernanda Solá-Ruiz

**Affiliations:** 1 Associated Professor, Prosthodontics Teaching Unit, Department of Stomatology, Faculty of Medicine and Dentistry, University of Valencia (Spain); 2 Teaching Assistant, Preventive Dentistry Teaching Unit, Department of Stomatology, Faculty of Medicine and Dentistry, University of Valencia (Spain); 3 Professor Assistant Doctor, Orthodontics Teaching Unit, Department of Stomatology, Faculty of Medicine and Dentistry, University of Valencia (Spain); 4 Full Professor, Prosthodontics Teaching Unit, Department of Stomatology, Faculty of Medicine and Dentistry, University of Valencia (Spain); 5 Teaching Assistant, Prosthodontics Teaching Unit, Department of Stomatology, Faculty of Medicine and Dentistry, University of Valencia (Spain)

## Abstract

**Background:**

To evaluate color changes to composite resins used to restore extracted teeth compared with composite discs after whitening with two agents: hydrogen peroxide (HP) and carbamide peroxide (CP).

**Material and Methods:**

Ten human molars with class V vestibular and palatine cavity preparation obturated with Vita hybrid nanocomposite were hemisected to obtain 20 specimens assigned randomly to two groups: O1 and O2. Twenty composite discs were divided into two groups: D1 and D2. The groups O1 and D1 were treated with 16% CP, while groups =2 and D2 were treated with 37.5 % HP. Chromaticity coordinates L*, a* and b* were registered using a spectrophotometer.

**Results:**

Statistically significant differences were found in O1 for L* and a*, in O2 for all three coordinates, and in D1 and D2 only for L*. Comparisons between groups found significant differences in ΔEe (end of treatment) between O1 and O2, between O2 and D2, and between D1 and O1.

**Conclusions:**

Both whitening agents produced significant decreases in the three-color components of composites used for dental restorations, while color changes to composite discs were limited to changes in luminosity. HP produced a greater color change to composite dental restorations than to composite discs.

** Key words:**In vitro study, whitening agents, hydrogen peroxide, and carbamide peroxide, dental restorations.

## Introduction

A basic criterion of contemporary dentistry is to perform resin composite dental restorations of the highest esthetic quality. At the same time, demand for tooth whitening treatments by patients is on the increase. In this context, composite materials are often exposed to the action of dental whitening agents, which undergo color changes when the material comes into contact with the bleaching products used (1-13).

Following light curing, composite resins experience what is known as a ‘dark reaction phase’. This phase has been estimated to last between 24 hours and 7 days until the composite reaches its definitive conversion level. When this phase is over and the material remains exposed to the oral medium, it is expected to show its definitive color (14). But as time passes the color can vary appreciably (15).

In the case of whitening agents, the whitening effect is due to pH, whereby the higher the concentration, the greater the oxidation process of resin composites and the color changes generated will be. Even a low concentration of HP can affect the color of photopolymerizable composites (3,9).

To evaluate the effects generated by whitening agents, CIELAB color space spectrophotometry is an accepted method of registering color parameters objectively (16-18), in which color difference is registered as ΔE, making it possible to register changes in the chromaticity and luminosity of teeth in clinical trials of dental whitening agents (9,19).

But few published studies have investigated color changes to composites used for dental restoration in situ in teeth after exposure to dental whitening agents, as most experiments have measured color changes using small blocks of resin in isolation.

For this reason, the aim of the present study was to evaluate color changes to composites used to restore extracted teeth generated by exposure to two whitening agents. The study hypothesis was that the whitening agents would not affect the color of the resin composite.

## Material and Methods

The study protocol was approved by the University of Valencia Ethics Committee for Research Involving Human Subjects (registration nº H14145265037779).

-Dental preparation 

Ten human molars were selected, extracted for orthodontic or periodontal reasons, free of caries and obturations, with intact enamel. After removing any remaining tissue and plaque, the teeth were stored in physiological serum. Two class V cavities were created in each tooth (one vestibular and the other palatine/lingual) with a cylindrical diamond bur with water cooling. The cavities had a square shape and were positioned in the middle third of the vestibular and palatine faces. The cavities measured 5 mm high, 5 mm wide and 2 mm deep, without beveling at the dig-superficial edges. The molars were then sectioned in mesial-distal direction with a diamond disc driven by a hand-piece, cutting each tooth into two halves (one vestibular, the other palatine), and cleaning the pulp chambers with a curette.

In order to distinguish between the two halves of teeth in each group, the apices were marked with different colored lacquers.

The enamel of the cavities was etched with 32% orthophosphoric acid gel (ScotchbondTM Universal Etchant. 3M, Minnesota, USA) for 15 seconds, washed with water for 15 seconds, and dried with a soft air jet. A single-step adhesive was applied as a single dose (ScotchbondTM Universal Adhesive. monodosis L-PopTM. 3M ESPE, Minnesota, USA) for 20 seconds, air-dried for 5 seconds, and photopolymerized with an LED lamp for 10 seconds. The cavities were obturated with a hybrid nanocomposite with A2 color (Ice. SDI, Victoria, Australia) applied in two 2 mm layers, and polymerized with the LED lamp for 40 seconds.

-Composite disc preparation 

Twenty identical composite discs were fabricated using a mold, of 2 mm thickness and 7 mm diameter. These were made from the same hybrid nanocomposite used to obturate cavities; each disc was polymerized for 40 seconds. When fabricated, the discs were stored in physiological serum.

Both dental specimens and discs were polished with polishing discs with three different abrasion grades (medium, fine, and superfine) (Sof-LexTM. 3M ESPE, Minnesota, USA) set in an angle-piece at low-speed without water cooling. Afterwards they were returned to storage in physiological serum.

 -Whitening agents 

Two whitening products were selected. The first was an in-office, chemo-activated agent, comprising 37.5% HP (PolaOffice +®. SDI. Victoria. Australia), applied by dual barrel syringe, as a blue-colored transparent gel with high viscosity and neutral pH. The second product, intended for domestic use, applied with a syringe, comprised 16% CP, a viscous transparent gel with neutral pH (PolaNight®. SDI. Victoria. Australia). Both were applied following the manufacturer’s instructions. In the case of composite dental restorations, the whitener was only applied to the dental crowns, leaving the roots free of the agent.

-Study groups 

Four groups were created (O1, O2, D1 and D2), each consisting of 10 specimens.

Group O1: 10 vestibular half-teeth treated with the CP whitening product for 90 minutes per day for 21 days, making a total exposure time of 31.5 hours.

Group O2: 10 palatine/lingual half-teeth treated with 37.5% HP whitening agent in three sessions of three 8-minute applications separated by intervals of one week, making a total exposure time of 72 minutes.

Group D1: 10 composite discs treated with 16% CP following the same procedure as Group O1.

Group D2: 10 composite discs treated with 37.5% HP following the same procedure as Group O2.

When the whitening procedures had come to an end, all samples were rinsed in water for 1 minute, and cleaned with a soft bristle tooth brush to remove any remaining whitening agent. Between whitening sessions until the end of the study period, all specimens were kept in physiological serum, changing the serum weekly.

-Color registration 

CIELab chromaticity coordinates L*, a* and b* of the composites used for dental restoration and composite discs were registered using the VITA Easyshade V spectrophotometer (VITA. Zahnfabrik, Germany). Color registration was performed at baseline, at the end of treatment, and one week after the end of treatment. To obtain comparable registers the same area of the composite was measured whether in dental restorations or as discs; each color evaluation was repeated three times consecutively, calculating mean values. The color parameters obtained were used to calculate the corresponding ΔE value. Reproducibility of the color evaluations was very high (intraclass correlation coefficient >0.9 for all three coordinates).

-Statistical analysis

Statistical analysis was performed using SPSS version 22.0 statistical software. Mean L*, a* and b* values and 95% confidence intervals (CI) were calculated. The Wilcoxon test was used to determine significant differences in L*, a* and b* values between baseline, end of treatment, and one week after end of treatment, in each test group. To identify differences between the four groups, the Mann Whitney U test was applied. Statistical significance was set at *p*<0.05.

## Results

 Table 1 shows comparisons of L*, a* and b* results between baseline and end of treatment in each group. In Group O1 (composite dental restorations treated with 16% CP) statistically significant differences were found for chromaticity coordinates L* (*P*<0.05) and a* (*P*<0.01). In Group O2 (composite dental restorations treated with 37.5% HP) very significant differences were found for all three chromaticity coordinates (*P*<0.01). But in Group D1 (composite discs treated with 16% CP) and Group D2 (composite discs treated with 37.5% HP), no statistically significant differences were found for coordinates a* and b* but significant differences were identified for coordinate L* in both groups.

**Table 1:**
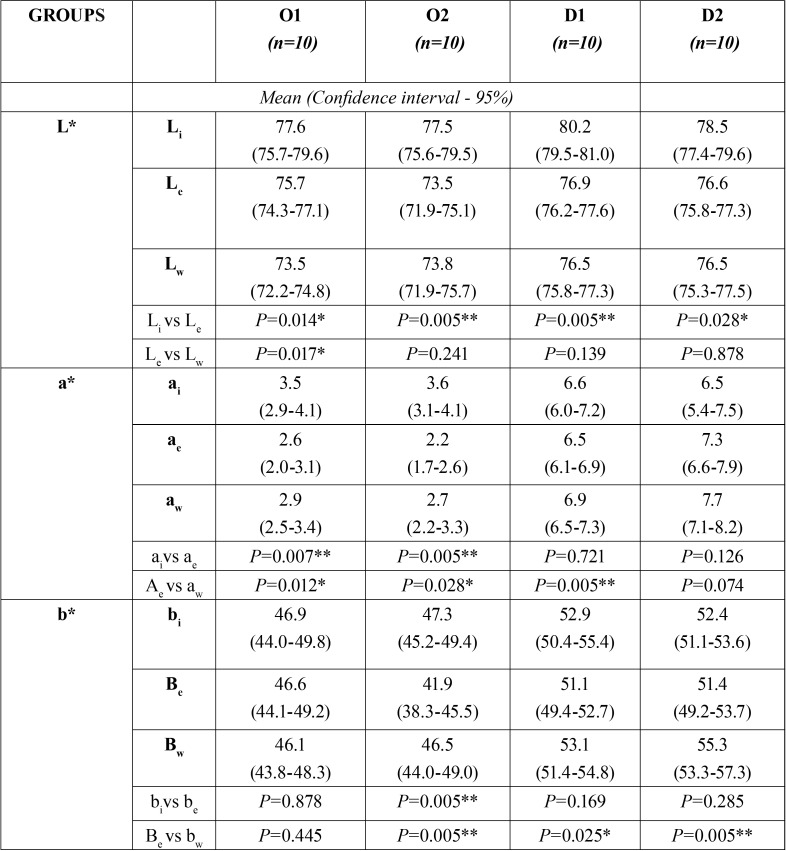
Chromaticity oordinates L*, a* and b* at baseline, end of treatment and one week after end of treatment in each group. i initial; e end of treatment; w one week after end of treatment; Wilcoxon test * *P*< 0.05; ** *P*< 0.01; O1= composite in dental restoration whitened with 16% carbamide peroxide; O2= composite in dental restoration whitened with 37.5% hydrogen peroxide; D1= composite disc whitened with 16% carbamide peroxide; D2= composite disc whitened with 37.5% hydrogen peroxide.

When L*, a* and b* values obtained at the end of treatment were compared with values registered one week later, no significant differences were found in coordinate L* in three of the groups ( Table 1), which indicates that luminosity remained stable after treatment ended, while Group O1 (*P*<0.05) lost luminosity beyond the end of treatment. Coordinates a* and b* generally showed statistically significant differences from the end of treatment to one week after end of treatment.

Comparisons of ΔEe and ΔEw values between the four groups are shown in  Table 2. Applying the Mann-Whitney test to compare these values, statistically significant differences (*P*<0.05) were found between Groups O1 and O2. Very significant differences (*P*<0.01) were found between Group O2 and D2. As for ΔEw, significant differences (*P*<0.05) were observed between Group D1 and O1.

**Table 2:**
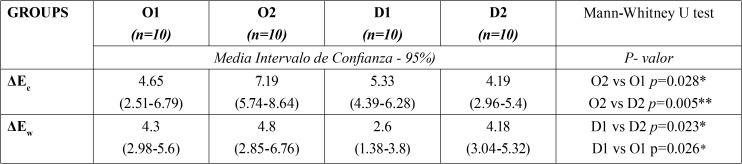
Intergroup comparison of ΔEe and ΔEw values; (Mann Whitney U test) * *P*< 0.05; ** *P*< 0.01.

## Discussion

Several authors have stated that color changes are more marked when the thickness of composite specimens is less than 3 mm (3,5,7). For this reason, the present study tested specimens, both composite dental restorations in teeth and discs, with a thickness of 2 mm. This made it possible to photopolymerize the obturations en bloc, a technique recommended when composite layers do not exceed 2 mm thickness (20).

The mechanism whereby resin composites change color when exposed to a whitening agent has not been clearly established. HP decomposes in water into oxygen and free radicals. The free radicals are responsible for whitening the teeth through the oxidation of the pigments responsible for their discoloration (12). But when composite materials are exposed to HP, these oxidant agents can destroy the composite’s resin matrix and cause discoloration as they affect the composite’s amine and unsaturated components (3,5). Moreover, peroxide free radicals probably cause oxidative excision of the polymer chains (21). The free radicals are eventually converted into water and oxygen, which facilitates the resin composite’s hydrolytic degradation process (9), and its discoloration. So, resin composites with higher resin content are more susceptible to degradation and therefore color changes (14).

Based on the results obtained in the present study, a general statistically significant decrease in post-treatment luminosity occurred in all groups, which continued to decrease during the week following the end of treatment in the group of composite dental restorations whitened with CP. This finding concurs with Anagnostou *et al.* (2) in which specimens also continued to lose luminosity after exposure to whitening agents.

Regarding the chromaticity coordinate a* (red-green), it was found that in the two groups of composite used for dental restoration a reduction in this coordinate was obtained at the end of treatment. But a week after the end of treatment all groups except the group of discs whitened with HP, underwent increases in a*.

As for the chromaticity coordinate b* (yellow-blue), only the group of composite dental restorations whitened with HP showed a reduction in this value at the end of treatment. One week after the end of treatment, all the groups obtained a decrease in this coordinate, except the group of composite dental restorations whitened with CP. These findings are similar to a study by Kamangar *et al.* (6) in which the b* coordinate increased in methacrylate-based resins after treatment with 15% CP and 40% HP. A study by Hubbezoglu *et al.* (5) also observed an increase in the b* coordinate for microhybrid and microfilled resin composites after exposure to 35% HP. For Mendes *et al.* (8), nanohybrid composite also showed a tendency towards yellowing after whitening with HP-based agents.

Analyzing ΔEe (end of treatment) values, it was found that HP (ΔEe = 7.2) produced greater color changes than CP (ΔEe = 4.7) to composite dental restorations. Differences were also found between dental restoration groups (ΔEe = 7.2) and composite discs (ΔEe = 4.2) whitened with HP at the end of treatment (it is accepted that most observers are able to perceive differences in Δ greater than 5). These results are similar to Kurtulmus-Yilmaz *et al.* (22), who whitened microcomposites and nanocomposites with 10% CP and 10% HP, obtaining ΔEe values of between 3.9 and 7.1, which were higher among specimens treated with HP.

In the groups whitened with CP, ΔEw (one week after end of treatment) was higher in the group of composite dental restorations (ΔEw = 4.3), than the group of composite discs (ΔEw = 2.6). But ΔEw in the group of composite discs treated with HP was higher (ΔEw = 4.18) in comparison with CP (ΔEw = 2.6).

The frequency of application of whitening products could contribute to a disparity in results between different studies. The present work set out to reproduce everyday clinical practice, following the schedules recommended by the manufacturers of the whitening products; during the intervals between applications, the specimens were stored in physiological serum. But in earlier studies, specimens were exposed to the whitening agents continuously (13,23).

The present study, like Li *et al.* (7), demonstrated that color changes to whitened teeth were significantly greater than to restoration materials in isolation. After whitening, dental restorations take on a different color from the teeth (5,7).

The results of the present study reject the initial hypothesis that whitening agents would not affect the color of the resin composite, as they were seen to alter, to greater or lesser degree, the color of the composites tested.

## Conclusions

Dental whitening agents, hydrogen peroxide and carbamide hydroxide, produce significant decreases in all three color components of resin composites used for dental restoration, while isolated composite discs only undergo changes in luminosity.

Hydrogen peroxide causes significantly greater color changes to composites used for dental restoration than composite disc specimens. This effect was not observed with carbamide peroxide.
